# Systematic Analysis of microRNA Biomarkers for Diagnosis, Prognosis, and Therapy in Patients With Clear Cell Renal Cell Carcinoma

**DOI:** 10.3389/fonc.2020.543817

**Published:** 2020-12-04

**Authors:** Guiyun Cheng, Mengru Li, Xiaoyu Ma, Fangmei Nan, Lu Zhang, Zhongyi Yan, Huimin Li, Guosen Zhang, Yali Han, Longxiang Xie, Xiangqian Guo

**Affiliations:** Cell Signal Transduction Laboratory, Bioinformatics Center, Henan Provincial Engineering Center for Tumor Molecular Medicine, Department of Preventive Medicine, School of Basic Medical Sciences, Institute of Biomedical Informatics, Henan University, Kaifeng, China

**Keywords:** clear cell renal cell carcinoma, miRNA, biomarkers, diagnosis, prognosis, molecular targeted therapy, circulating miRNA

## Abstract

The ever-increasing morbidity and mortality of clear cell renal cell carcinoma (ccRCC) urgently demands updated biomarkers. MicroRNAs (miRNAs) are involved in diverse biological processes such as cell proliferation, differentiation, apoptosis by regulating their target genes’ expression. In kidney cancers, miRNAs have been reported to be involved in tumorigenesis and to be the diagnostic, prognostic, and therapeutic response biomarkers. Here, we performed a systematic analysis for ccRCC-related miRNAs as biomarkers by searching keywords in the NCBI PubMed database and found 118 miRNAs as diagnostic biomarkers, 28 miRNAs as prognostic biomarkers, and 80 miRNAs as therapeutic biomarkers in ccRCC. miRNA-21, miRNA-155, miRNA-141, miRNA-126, and miRNA-221, as significantly differentially expressed miRNAs between cancer and normal tissues, play extensive roles in the cell proliferation, differentiation, apoptosis of ccRCC. GO and KEGG enrichment analysis of these miRNAs’ target genes through Metascape showed these target genes are enriched in Protein Domain Specific Binding (GO:0019904). In this paper, we identified highly specific miRNAs in the pathogenesis of ccRCC and explored their potential applications for diagnosis, prognosis, and treatment of ccRCC.

## Introduction

Renal cell carcinoma (RCC) contributed by malignant proliferation of the tubular epithelial cells is the most common malignant tumor in the kidney (1). Nearly 209,000 people are diagnosed with kidney cancer and nearly 102,000 patients die from it worldwide each year (2). Clear cell renal cell carcinoma (ccRCC) is the most prevalent histological subtype of RCC (3), accounting for nearly 80% of RCC (2). In the early stage, ccRCC is usually asymptomatic or insidious (4), with low malignant degree and relatively slow tumor progression (5). The ccRCC tumor cannot be easily detected until it grows to a certain size (6). Moreover, ccRCC patients are inevitably faced with high metastasis risk and chemotherapy resistance (1). Currently, nearly 30% of patients are diagnosed with metastasis (7). For ccRCC patients with advanced stage or cancer recurrence, several molecule-targeted drugs including sunitinib, sorafenib, and aldesleukin have been used as the clinical first-line therapy (8), which has greatly improved the patients’ survival time compared with chemo-radiotherapy. However, due to molecular heterogeneity and different sensitivity to drugs between patients, median disease-free and overall survival times of patients remain short. Therefore, more novel effective diagnostic and prognostic biomarkers need to be developed for ccRCC patients to guide personalized therapies (3, 9).

Recently, molecular biomarkers have been developed for early diagnosis, treatment response prediction, and prognosis in ccRCC (10, 11). MicroRNAs (miRNAs), which are small non-coding RNAs and usually consist of 19–25 nucleotides in length (12), can target the downstream genes and regulate their expression (13). The 3′ untranslated region of mRNAs is the common target of miRNAs (14), and their binding can contribute to degrading mRNA or inhibiting protein translational process (7). LncRNAs are also found as targets of miRNAs (15). Many studies have shown that miRNAs are abnormally expressed in diverse types of human cancers (15–18) and involved in varieties of biological processes, from angiogenesis in tumor microenvironment to tumor distant metastasis (19). For instance, overexpressed miR-21 can promote malignant cell proliferation in head and neck squamous carcinoma cell lines by regulating the expression of programmed cell death 4 (PDCD4) gene (20). Overexpression of miR-126 can accelerate gastric carcinogenesis by suppressing sex-determining-region Y-box2 (SOX2) (21). In human renal cancer, many abnormally expressed miRNAs including miR-21, miR-155, miR-126, miR-221, and miR-141 were found to be involved in the process of carcinogenesis and tumor suppression (5, 10, 21–23). These results show that miRNAs may play crucial roles in ccRCC and possibly act as useful tools for diagnosis, prognosis, and target treatment of patients (22).

Over the last years, a novel diagnostic technology called as “liquid biopsy” has appeared with surge of interest. Liquid biopsy avoids invasive procedures and can be used to detect tumor cells or nucleic acids in body fluids repeatedly (24). For different cancer patients, studies have found tumor-specific or related changes of circulating nucleic acids such as DNA, mRNA, and miRNA in peripheral blood (25). Among them, acquisition of circulating miRNAs through plasma or serum not only can monitor the presence of early tumors, but also indicate the status and dynamics of late tumors, as well as tumor recurrence and drug sensitivity in real time (26, 27). Therefore, using circulating miRNAs collected from biological fluids without invasive procedures may be a prospective liquid biopsy in ccRCC patients’ management.

In this paper, we systematically summarized those tumor-specific aberrantly expressed miRNAs and circulating miRNAs respectively as high-efficient biomarkers and more critically evaluated their roles in the diagnosis, treatment response prediction, and prognosis of ccRCC patients.

## Materials and Methods

This review was performed according to the PRISMA (Preferred Reporting Items for Systematic reviews and Meta-Analysis) (28) and PICOS (Population, Intervention, Control, Outcome, Study Design) guidelines (29). The detailed criteria of selection were as follows: PRISMA guideline according to PRISMA 2009 checklist (**Table S1**) and PICOS guideline (http://www.prisma-statement.org/PRISMAStatement/) (**Table S2**).

### Selection Criteria of Published Studies

To identify the published microRNA biomarkers for diagnosis, prognosis, and therapy in patients with clear cell renal cell carcinoma, entry terms of “(non-coding RNA) OR (miRNA OR microRNA OR miR) AND (Clear cell renal cell carcinoma OR ccRCC)” were firstly searched in the NCBI PubMed database.

A total of 1,410 studies were obtained after removing any duplication of article content. Then we excluded some studies rigorously according to our criteria. The criteria of record exclusion were as follows: i) Reviews and meta-analysis and bioinformatics; ii) Case report; iii) Article not in English; iv) No miRNA study; v) Non-human study: cell lines or/and mice; vi) Other disease. And the criteria of full text articles’ exclusion were also listed as follows: i) Not primary ccRCC; ii) Comparison of microRNA expression in FF *vs* FFPET blocks; iii) Methylation or polymorphism; iv) Not analysis of phenotype of interest. Finally, 181 intratumor miRNAs and 13 circulating miRNAs in ccRCC were collected from 126 and 10 studies, respectively.

### Screening Strategies of Potential Target Genes

The selection of potential target genes was predicted by TarBase v7.0 (30), Target Scan Human (31), microT-CDS database (32). Venny 2.1.0 (https://bioinfogp.cnb.csic.es/tools/venny/index.html) was used to select the common target genes in three databases. Gene Ontology (GO) and Kyoto Encyclopedia of Genes and Genomes (KEGG) enrichment analyses were performed in Metascape (33).The Network of enriched terms and Boxplot of miRNAs’ corresponding target genes were constructed within *P* value cut-off 0.01. Cytoscape was used to construct a network related with cellular functions of miRNAs in tumorigenesis (28).

### Statistical Analysis

Hazard ratios (HRs) estimated by the Cox proportional-hazards was applied in the evaluation of each prognostic model or panel. We also made Forest plots of miRNAs in prognostic biomarkers for predicting the risk.

## Results

**Figure 1:**
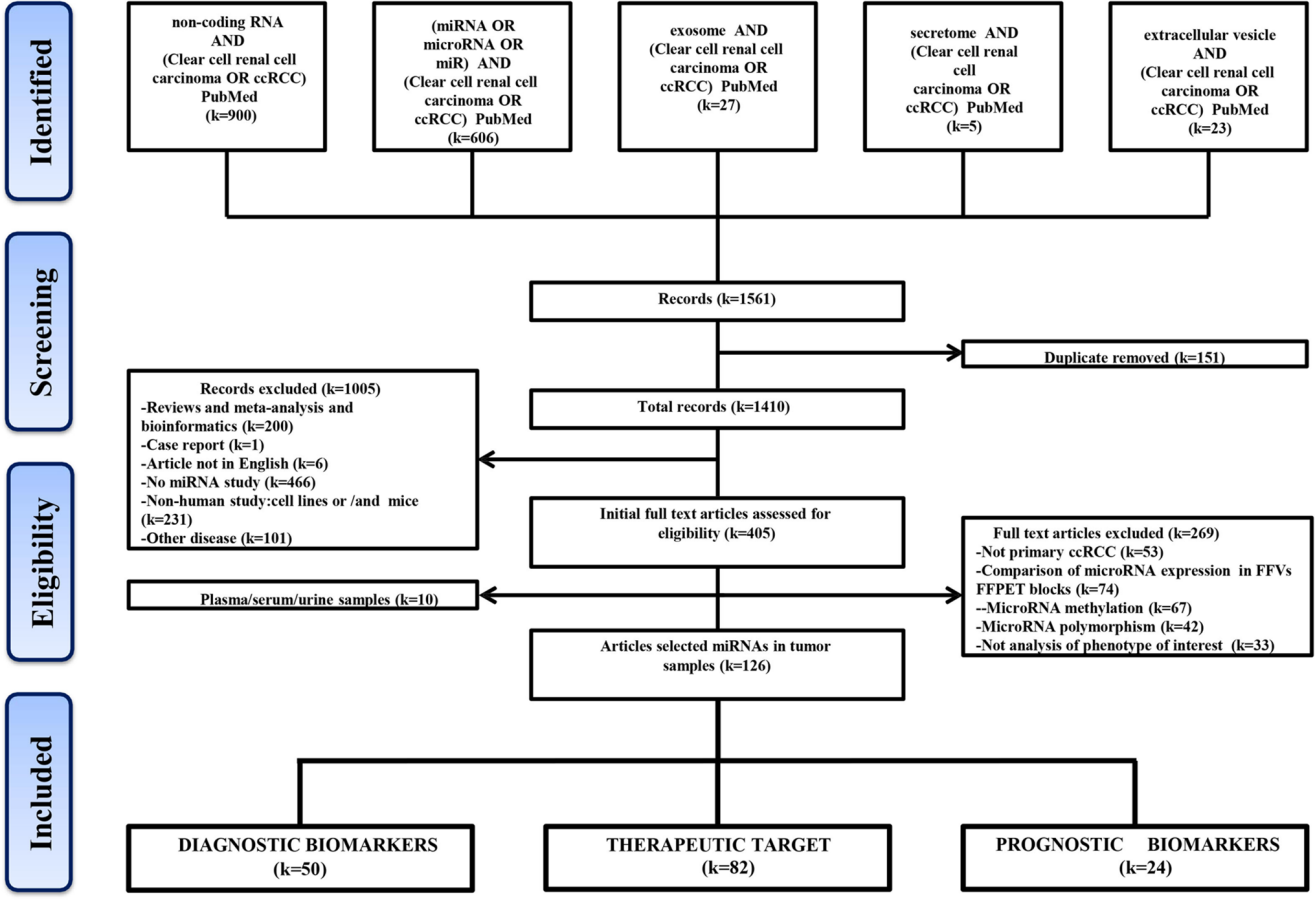
Flow-chart diagram of this study. k = number of literature records.

A flow chart shows the process of identification, inclusion, and exclusion (**Figure 1**). The initial records collected 1,410 papers after removing the duplicates in the 1,561 papers from PubMed Database. Through browsing titles and abstracts, 405 papers were left by exclusion criteria described in **Figure 1**. Further, according to the availability of full text, 10 studies were involved circulating miRNAs, and the remaining 126 articles about tissue-related miRNAs in diagnosis, prognosis, and treatment were finally preserved. These included 50 articles reporting miRNAs as potential ccRCC diagnostic biomarkers, 82 in target treatment, and 24 in prognosis.

### Intratumor miRNAs Act as the Diagnostic Biomarkers in ccRCC

Fifty articles reporting miRNAs as diagnostic biomarkers were screened out and ultimately revealed 118 differentially expressed miRNAs in ccRCC tissues compared with adjacent normal tissues (**Table S3**), including 48 upregulated miRNAs and 70 downregulated miRNAs in ccRCC tissues. In addition, two miRNAs (miR-126 and miR-30b) showed opposite expression results in two studies respectively. Abnormally expressed miRNAs reported in more than two studies with similar results were specifically summarized in **Table 1**. miR-21 was the most frequently investigated in eight studies, which was upregulated in seven studies, while only one contradicted. Five studies showed that miR-155 was consistently upregulated in tumor tissues compared with normal tissues. In addition, miR-221, miR-122, and miR-210 were reported to be upregulated in two studies, while both miR-144 and miR-144-3p were downregulated in tumors from two studies. Szabo et al. found that 79.2% of the ccRCC specimen displayed miR-21 and miR-221 coexpression, in contrast to 33.3% of the normal renal tissue being observed coexpression (10). To investigate the specific role of upregulated miR-21 for ccRCC cell proliferation and apoptosis, Cao et al. demonstrated that overexpressed miR-21 facilitated proliferation and inhibited apoptosis *via* flow cytometric analysis and MTT array (7). Fan et al. found that markedly increased miR-122 exerted its function of promoting ccRCC metastasis through directly targeting Dicer, implying that miR-122 may be used for differentiating ccRCC with ability of underlying metastasis (34). These significantly upregulated or downregulated miRNAs have the potential to be developed as a diagnostic marker when further validated.

**Table 1:** miRNAs as diagnostic biomarkers for ccRCC in more than two studies.

Name	Expression Level^§^	n (ccRCC)	n (Control)	Detection Method	Target	Sample source	PubMed ID
miR-21	**Up**	104	104	qPT-PCR	TIMP3	Tissue	29131259
	**Down**	30	10	qPT-PCR	ND	Fresh frozen	24129247
miR-155	**Down**	30	10	qRT-PCR	ND	Fresh frozen	24129247
	**Up**	137	77	qRT-PCR	ND	Tissue	23050614
	**Up**	78	78	qRT-PCR	ND	Fresh frozen	24647573
	**Up**	30	10	qRT-PCR	ND	Fresh frozen	24129247
	**Up**	32	132	qRT-PCR	ND	Fresh frozen	25381221
miR-141	**Down**	30	10	qRT-PCR	ND	FF Fresh frozen	24129247
	**Down**	78	78	qRT-PCR	EphA2	Fresh frozen	24647573
miR-221	**Up**	28	28	qRT-PCR	TIMP2	Tissue	26191221
	**Up**	24	24	qRT-PCR	ND	Tissue	27427222
miR-122	**Up**	32	32	qRT-PCR	ND	Fresh frozen	25381221
	**Up**	148	60	qRT-PCR	Dicer	Fresh frozen	28921581

^§^Down, signiﬁcantly downregulated miRNAs in human ccRCC tissues; Up, signiﬁcantly upregulated miRNAs in human ccRCC tissues; qRT-PCR, quantitative real-time PCR; ND, not determined; CASC2, cancer susceptibility 2; EphA2, erythropoietin-producing hepatocellularA2; TIMP2, TIMP metallopeptidase inhibitor 2.

### Intratumor miRNAs as the Prognostic Biomarkers in ccRCC

The correlation between miRNAs and prognosis of ccRCC patients was analyzed in 24 articles, showing that 28 miRNAs were associated with overall survival (OS) or cancer specific survival (CSS) or progression free survival (PFS) for ccRCC patients (**Table S4**). In these studies, the researchers utilized qRT-PCR to measure miRNA expression and demonstrated that miRNAs were effective and efficient predictors of OS and CSS in ccRCC patients by using Kaplan–Meier curve and multivariable Cox regression. Among them, the expression level of 16 and 12 miRNAs in ccRCC showed a favorable and unfavorable prognosis, respectively. In particular, three miRNAs including miR-122, miR-126, and miR-21 were identified as potential prognostic biomarkers in ccRCC in more than two studies (2, 10, 21, 22, 35) (**Table 2**). miR-122 was shown as an unfavorable prognostic marker in ccRCC patients (10, 35).

**Table 2:** miRNAs as prognostic biomarkers for ccRCC in more than two studies.

Name	Expression Level	n (ccRCC)	Sample source	Detection Method	Prognosis^§^	Survival	PubMed ID
miR-21	**Up**	37	Formalin-fixed paraffin-embedded	qRT-PCR	Unfavorable	CSS	25279769
	**Up**	71	Tissue	qRT-PCR	Unfavorable	OS and DFS	22580180
miR-126	**Down** **Down** **Down**	264 37 116	Tissue and Formalin-fixed paraffin-embedded Formalin fixed paraffin embedded Formalin fixed paraffin embedded	qRT-PCR qRT-PCR qRT-PCR	Favorable Favorable Favorable	OS and DFS CSS DSS and RFS	25572155 25279769 30988818
miR-122	**Up** **Up**	46 80	Tissue Fresh frozen	qRT-PCR qRT-PCR	Unfavorable Unfavorable	MFS PFS	30483771 28534944

^§^CSS, cancer specific survival; OS, overall survival; DFS, disease free survival; DSS, disease specific survival; RFS, recurrence free survival; MFS, metastasis free survival; PFS, progression free survival.

Fan et al. revealed that upregulated miR-122 promoted proliferation and invasion of ccRCC by inhibiting FOXO3 (Forkhead box O3) and was attached to shorter metastasis-free survival time as a tumor promoter (34). Additionally, overexpression of miR-122 was reported to induce cell cycle arrest and inhibit cell apoptosis in HCC (hepatocellular carcinoma) (36). Qin et al. showed that increased miR-122 inhibited cell invasion and migration in NSCLC (non-small-cell lung cancer) (37). However, overexpressed miR-122 displayed an unfavorable prognostic subtype as an oncogene in CRC (colorectal cancer) (38). These data collectively show that miR-122 may serve as a tumor promoter or suppressor in various types of cancers. Studies had demonstrated that miR-126 was upregulated in ccRCC, but its expression was decreased along with increasing grade and advanced tumor stage (39–43). Similarily, Carlsson et al. found lower expression of miR-126 was significantly associated with shorter time to recurrence of ccRCC (41). The expression level of other 25 miRNAs also showed significant associations with ccRCC patients’ survival.

In the prognostic miRNAs we collected, only two miRNAs including miR-21 and miR-126 with HR and CI value were found in more than two studies; the other miRNAs were just found in one study. To predict the risk, we performed a meta-analysis of prognostic impact on miR-21 and miR-126 by using the random effects model. The Forest plot results showed that miR-21 (HR = 2.67, 95%CI [1.51–4.71]) and miR-126 (HR=0.46, 95%CI [0.29–0.73]) (**Figure S1**) expression were significantly associated with survival of ccRCC, respectively.

### Intratumor miRNAs Are Related With Molecular Targeted Therapy and Treatment Response in ccRCC

**Figure 2:**
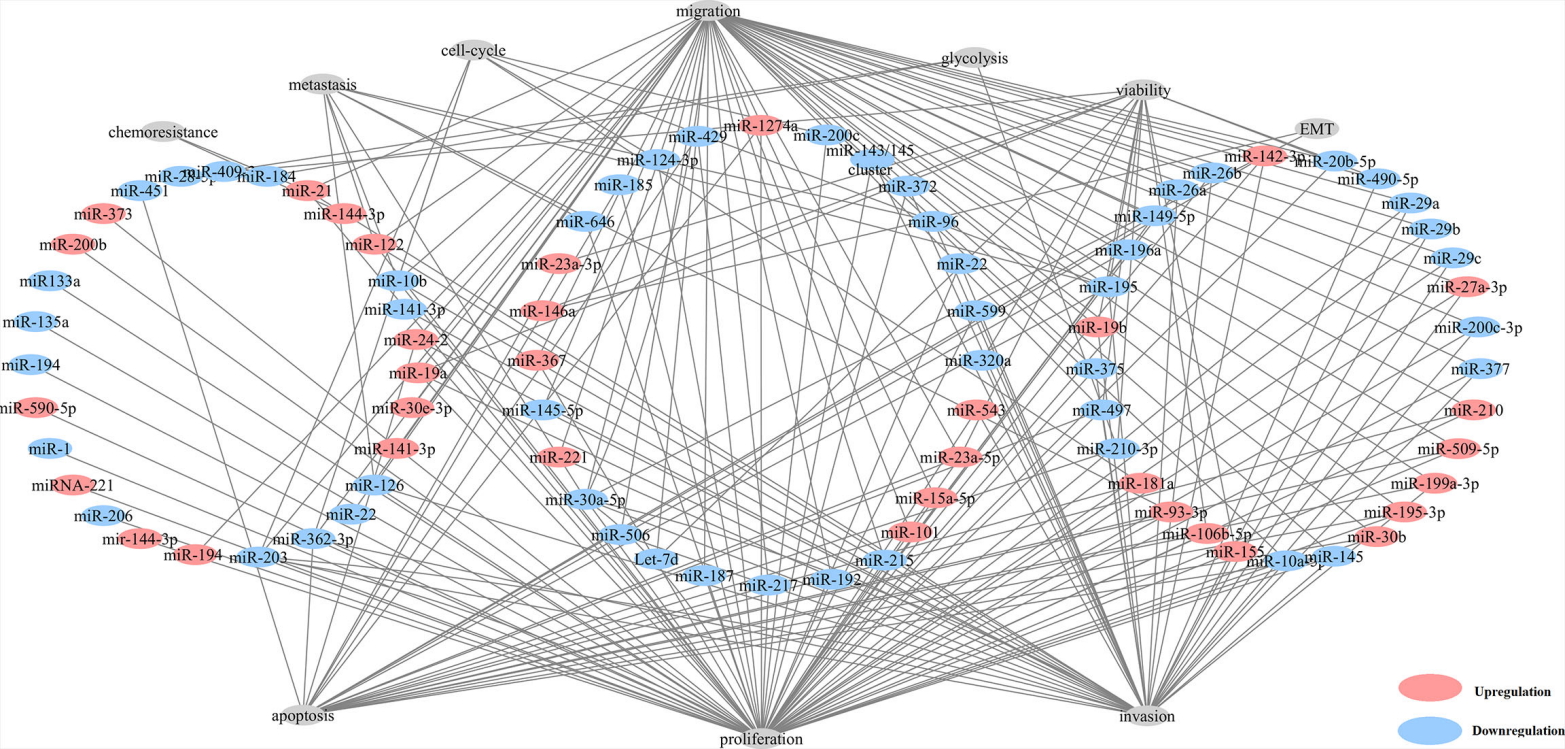
Network of aberrantly expressed miRNAs in diverse cellular functions.

Here, we found that 80 aberrantly expressed miRNAs in 82 studies were related with tumorigenesis (**Figure 2** and **Table S5**). The majority of these miRNAs could result in neoplastic proliferation. miR-200c (39), miR-181a (44), miR-195 (45) and miR-362-3p (46), can regulate cell cycle, proliferation, and apoptosis, in control of the cellular atypia during neoplasia. miR-122 (35, 47), miR-10b (44), miR-195 (45) and miR-30a-5p (45) contributed to architectural atypia, prognosis, invasion, and distant metastasis of tumors. Besides, miR-21 induced cell migration, invasion (15), and chemoresistance for anti-tumor agents of paclitaxel, fluoropyrimidine 5-FU, oxaliplatin, sunitinib in ccRCC tumors (15).

miR-144-3p, miR-200c, miR-99b-5p, and miR-21 were found as response biomarkers for drug treatment (**Table S6**). Sunitinib, the first-line therapy for advanced ccRCC, is a multitargeted receptor tyrosine kinase (RTK) inhibitor (48). miR-144-3p can promote sunitinib sensitivity by downregulating ARID1A (49). miR-21 can increase both gefitinib and sorafenib sensitivity (48). Two studies had showed the important therapeutic function of miR-21 (48, 50). miR-200c targets heme oxygenase-1 and sensitizes ccRCC cell lines to sorafenib (51). miR-99b-5p is a remarkable TKI treatment candidate (52). Inhibitors of phosphoinositide 3-kinase (PI3K)/Akt pathway may be effective therapeutic drugs for ccRCC. PTEN is a strong negative regulator of this pathway and also a direct target of miR-21 (48). Tumor suppressor metformin (MF) can also inhibit AKT/mTOR pathway by activating AMPK, which implied that MF treatment obviously relied on miR-21 expression (50).

### Biological Function Analysis of Circulating miRNAs in ccRCC

Circulating miRNAs from liquid biopsy are considered as one of the valuable tumor-specific biomarkers which can be easily and steadily detected and indicate the status of the tumor. Here, 13 circulating miRNAs in 10 studies were carefully screened out (**Table 3** and **Table 4**). miR-210 (53), miR-1233 (53, 54), miR-508-3p (55), miR-885-5p (55), miR-34a (54), miR-141 (54), miR-144-3p, and miR-210-3p (56) were reported to be diagnostic biomarkers, as well as miR-122-5p (57), miR-206 (57), miR-150 (58) and miR-224 (59) were treated as prognostic biomarkers.

**Table 3:** Circulating miRNAs as diagnostic biomarkers for ccRCC.

Name	Expression Level	n(ccRCC)	n(control)	Sample source	PubMed ID
miR-210	**Up** **Up**	82 34	80 23	Serum exosomes Serum	28753793 24212760
miR-1233	**Up**	82	80	Serum exosomes	28753793
	**Up**	30	15	Serum	28336290
miR-508-3p	**Down**	85	10	Serum	31661117
miR-885-5p	**Up**	85	10	Serum	31661117
miR-34a	**Down**	30	15	Serum	28336290
miR-141	**Down**	30	15	Serum	28336290
miR-144-3p	**Up**	106	123	Plasma	27633984
miR-625-3p	**Down**	50	74	Serum	31737199
miR-210-3p	**Up**	38	10	Urine	29050224

**Table 4:** Circulating miRNAs as prognostic biomarkers for ccRCC.

Name	Expression Level	n (ccRCC)	Prognostic value	Survival event	Sample source	PubMed ID
miR-122-5p	**Down**	68	Unfavorable	PFS, CSS, and OS	Serum	29410711
miR-206	**Down**	68	Unfavorable	PFS, CSS, and OS	Serum	29410711
miR-150	**Down**	94	Favorable	RCC-specific survival	Plasma	28639257
miR-224	**Up**	108	Unfavorable	PFS	Serum exosomes	29299115

miR-625-3p served as a diagnostic and prognostic biomarker (60). Zhang et al. reported that exosomal miR-210 and miR-1233 from serum samples in different stages of ccRCC were significantly upregulated compared to healthy individuals, and their expression levels after receiving tumor resection were also obviously lower, which manifested serum exosomal miRNAs might have specific diagnostic implications (53). Zhao et al. found that miR-625-3p was decreased in serum of ccRCC patients and can differentiate ccRCC patients from healthy controls (60). Increased miR-625-3p was found to be associated with lower overall survival in ccRCC patients. Furthermore, miR-625-3p can promote the migration and invasion of ccRCC and inhibit the apoptosis *in vitro* (60). Kirschner et al. confirmed that miR-625-3p in plasma/serum and tumor specimens from Malignant Pleural Mesothelioma (MM) patients were both upregulated and revealed that miR-625-3p was a promising diagnostic biomarker for MM (60).

### Well-Known Biological Processes of ccRCC Associated With Dysregulated miR-21

**Figure 3:**
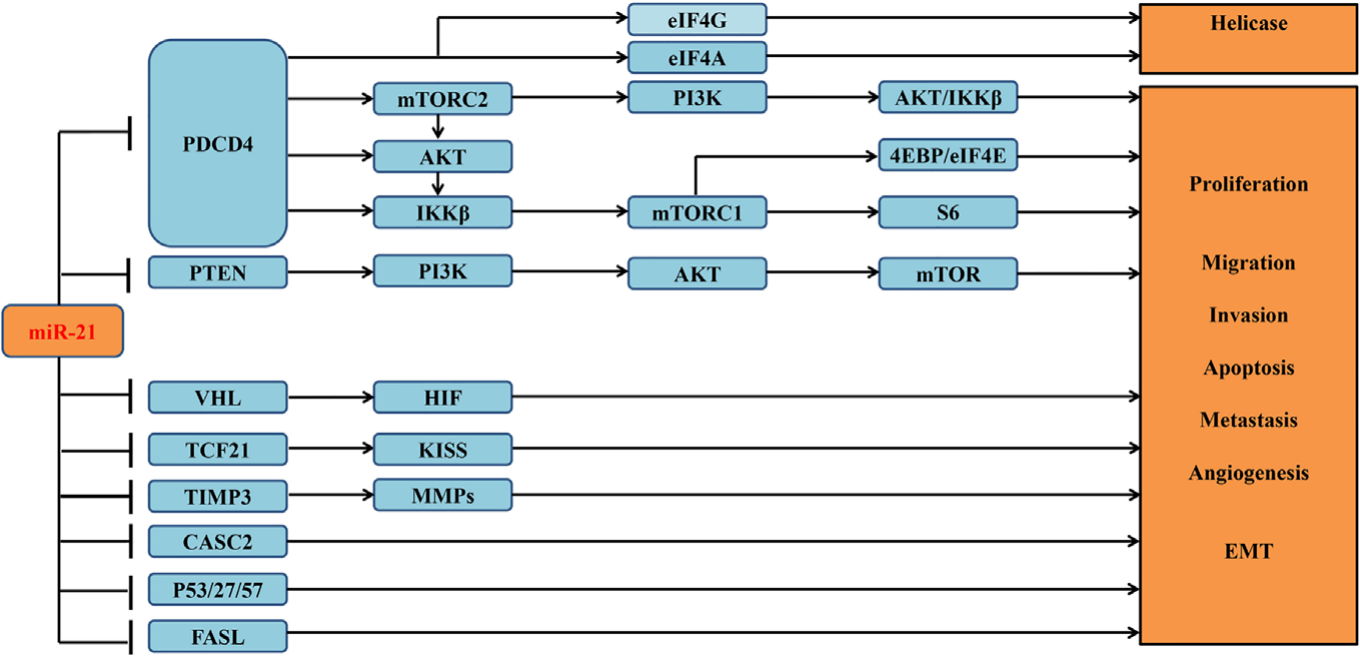
Verified downstream target genes and involved signaling pathways associated with miR-21 in the tumorigenesis of ccRCC.

miRNAs are reported to be involved in a variety of signaling pathways (19). Previous study had reported that miR-21 is highly expressed in ccRCC tumor tissues and cell lines (10). And its overexpression can attenuate multiple downstream apoptosis-related genes, including programmed cell death 4 (PDCD4) (13), gene of phosphate and tension homology deleted on chromosome ten (PTEN) (10), and death receptor gene (FASL) (10). Overexpressed miR-21 can decrease the expression of *PDCD4* through regulating mRNA translation initiation factors to suppress helicase activity and then contribute to the malignant transformation of anti-apoptotic cells (13). miR-21 can also inhibit other tumor suppressor genes associated with cell cycle in addition to *PDCD4* (2) (**Figure 3**), whose downstream cascades of enzymatic reactions are activated or inactivated. Ultimately, abnormal signaling pathways can induce tumor angiogenesis and progression. Overexpressed miR-21 can also modulate several lncRNAs to involve in the proliferation of tumor cells (15). Recent study concentrated on lncRNA–miRNA–mRNA regulatory network (9), which may be a major step forward in the search for a cure for ccRCC.

### Potential Signal Pathways Regulated by miRNAs

**Figure 4:**
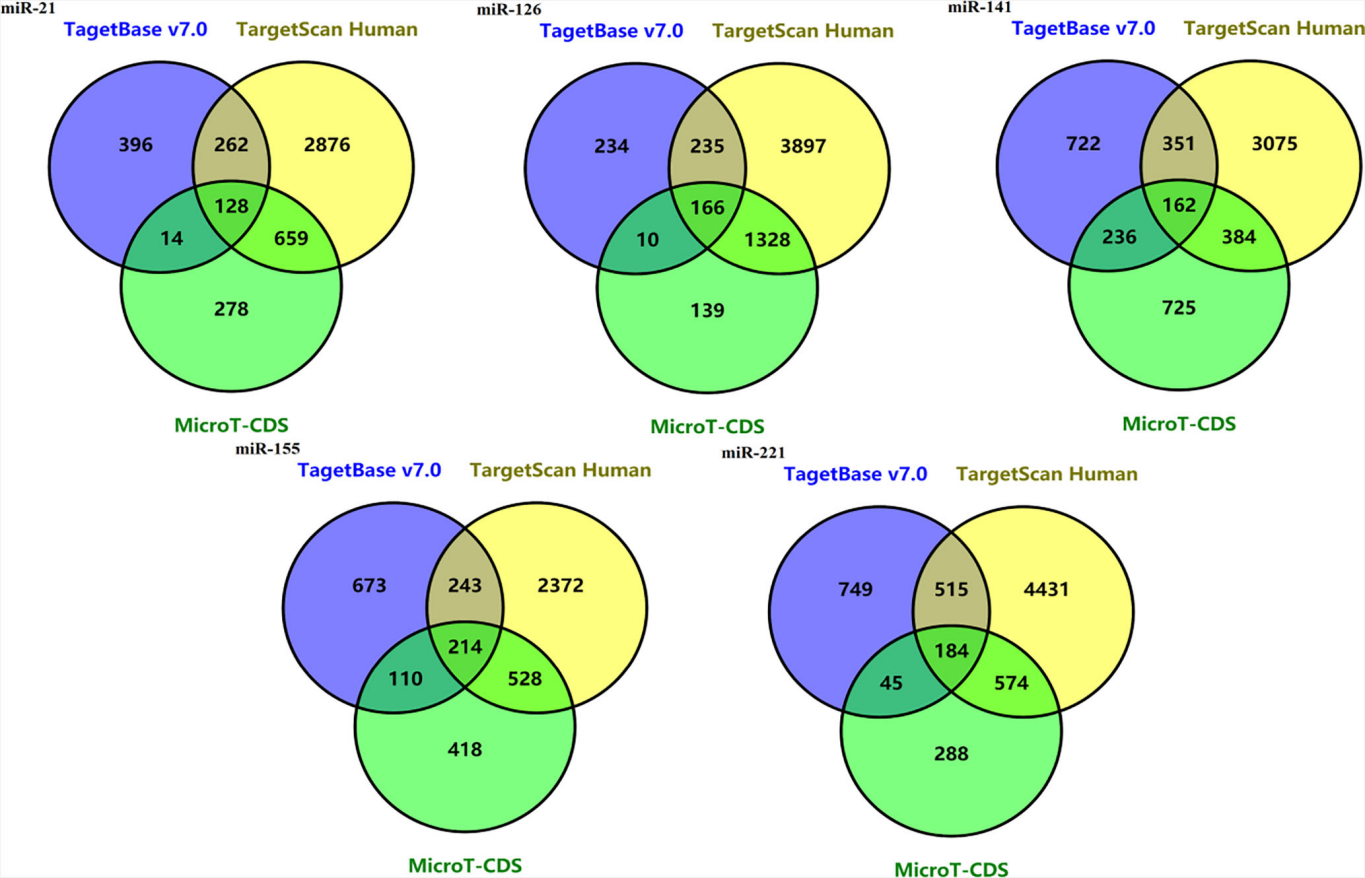
Venn diagrams of potential target genes for five miRNAs including mir-21, miR-221, miR-141, miR-155, and miR-126 from three predicting tools.

128 potential target genes for miR-21 were identified in three databases (TarBase v7.0, Target Scan Human and microT-CDS database). Target gene numbers of other four miRNAs including miR-221, miR-141, miR-155, and miR-126 were 184, 162, 214, and 166, respectively (**Figure 4**).

**Figure 5:**
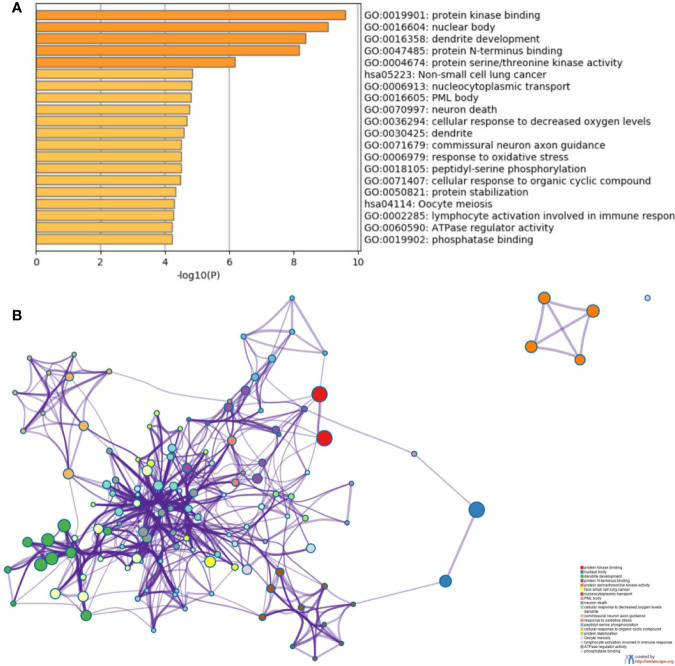
Functional enrichment analysis of the potential target genes of miRNA-21 **(A)**. Chart of the GO and KEGG enriched pathways **(B)**; Network of GO and KEGG enriched terms colored by clusters. PDCD4, programmed cell death 4; mTOR, mammalian target of rapamycin; mTORC1, mTOR complex 1; mTORC2, mTOR complex 2; eIF4A and eIF4G, RNA translation initiation factors; PI3K, phosphoinositide 3-kinase; AKT, Ser/Thr protein kinase; IKK*β*, one of the catalytic subunits in the IKK complex can activate the nuclear transcription factor; 4EBP/eIF4E, Von Hippel–Lindau disease tumor suppressor; S6, ribosomal proteins 6 kinases; PTEN, gene of phosphate and tension homology deleted on chromosome ten; VHL, Von Hippel–Lindau gene; HIF, hypoxia-inducible transcription factors; TCF21, Transcription factor; KISS, a member of the metastasis suppressor family; TIMP3, TIMP metallopeptidase inhibitor 3; MMPs, matrix metalloproteinases that can facilitate tumor growth, invasion and metastasis by degrading extracellular matrix; CASC2, long non-coding RNA and tumor suppressor gene; P27/57, cell cycle proteins; FASL, death receptor gene; EMT, epithelial-to-mesenchymal transition.

To further understand the biological processes these target genes may get involved, Metascape was performed for GO and KEGG enrichment analysis (**Figure S2**–**S5** and **Figure 5**). The enrichment analysis showed that the target genes of miR-21 and miR-155 may be involved in both Phosphatase Binding (GO:0019902) and Peptidyl-serine Phosphorylation (GO:0018105); the target genes of miR-155 and miR-141 may be involved in both Transferase Complex (GO:1990234) and Wnt signaling pathway (GO:0016055); the target genes of miR-155 and miR-221 may be involved in both Myeloid Cell Differentiation (GO:0030099) and Cellular Response to Hormone Stimulus (GO:0032870); the target genes of miR-221 and miR-141 may be involved in RNA Polymerase II Regulatory Region Sequence-specific DNA Binding (GO:0000977). Finally, the target genes of miR-126, miR-141, miR-221, miR-155 may all participate in Protein Domain Specific Binding (GO:0019904). These results suggested the downstream targets regulated by abnormally expressed microRNAs may contribute to diverse biological processes in ccRCC.

## Discussion and Conclusion

ccRCC is the most common subtype of RCC with poor prognosis and accounts for nearly 80% of RCC. miRNAs are small non-coding RNAs which mainly function at the post transcriptional levels. Recent studies have revealed that miRNAs participate in tumorigenesis and metastasis (10, 19). Detecting these abnormally expressed miRNAs and ascertaining their roles can also assist cancer diagnosis and treatment, which is superior to routine detection. In this paper, we summarized and classified the differentially expressed miRNAs in ccRCC tissues through literature research. These miRNAs may be used as potential biomarkers of ccRCC in the future.

121 differentially expressed miRNAs were obtained from fifty articles. Notably, miR-21 was the most broadly identified miRNA with highest frequency of significances in seven studies and was shown upregulated in ccRCC. It was also found to be upregulated in many other tumors consistently, such as lung cancer (61), B-cell lymphoma (62), gastric cancer (63), prostate cancer (64), colorectal cancer (65), and ampullary adenocarcinoma (66). This implies that miR-21 expression is significantly associated with cancer. In addition, upregulated miR-21 can be used to distinguish ccRCC, PRCC (papillary renal cell carcinoma) from other subtypes (67). It had also been shown that miR-21 upregulation was significantly related to gender, metastasis, stage, and lymph node status (2). A series of reported target genes of miR-21 were summarized in this paper (**Figure 3**). By suppressing these genes, miR-21 can affect cell proliferation, invasion, migration, and EMT in ccRCC (7). In a word, miRNA-21 may be used as a promising diagnostic marker and can be broadly applied to patients’ clinical treatment guidance.

Moreover, 28 miRNAs from 24 different studies were reported as putative prognostic biomarkers for ccRCC. Three of these miRNAs (miR-21, miR-122 and miR-126) studied in more than two studies were used for further analysis. miR-21, miR-122 and miR-126 were found to be significantly up-regulated in ccRCC tumor samples (10, 22, 41, 68). Upregulated miR-21 and miR-122 were associated with poor overall survival or cancer specific survival or progression-free survival (22, 35, 69), while downregulated miR-126 was an unfavorable biomarker for metastatic ccRCC (40).

Metformin, sunitinib and sorafenib are common drugs used to treat RCC (48, 50). It has been demonstrated that miR-21 expression can be induced by metformin (50). miR-144-3p and miR-21 expression can affect cell sensitivity to these drugs (50, 70). It has been reported that miR-21-silencing can decrease multiple drug-resistant gene expression and promote the chemo-sensitivity in ccRCC (48). Based on these studies, miR-21 may be an important therapeutic target. These data indicate that miR-21 may be the diagnosis, treatment, and prognosis biomarker candidate of ccRCC. It is imperative for us to perform experimental verification to make a thorough inqury about the clinical utility of miR-21. However, our study is a bioinformatic study without any experimental results. Hence, it is better to perform experiments in an *in vitro* model of ccRCC to study the effects of miR-21 on proliferation, invasion, and response to TKIs. To demonstrate clearly the involvement of miRNA on the prediction of response to therapy, studies *in vivo* were also required. It should be wonderful if defining a miRNA different from miR-21 (already well known in the tumorigenic processes) is able to predict the prognosis and response to therapy.

Metastasis is extremely common in ccRCC, and increasing studies can pave the way to the clinical use of miRNAs as prognostic markers for metastasis. Through searching literature, we found three published articles about prognostic miRNAs for ccRCC metastasis (**Table 5**). miR-122 and miR-30a were found to be the prognostic biomarkers of ccRCC metastasis. Increased miR-122 in patients has poorer metastasis-free survival rates in two articles (71, 72). Decreased miR-30a was associated with hematogenous metastasis and shorter metastasis-free survival (73). Further investigations are urgently needed to identify metastatic *vs* non-metastatic miRNAs for ccRCC patients.

**Table 5:** Metastasis-related miRNAs as prognostic biomarkers for ccRCC.

Name	Expression Level	n (ccRCC)	Target	Sample source	PubMed ID
miR-122	**UP**	148	Dicer	Fresh frozen	28921581
	**UP**	46	FOXO3	Tissue	30483771
miR-30a	**Down**	90	DLL	Tissue	23826258

Circulating miRNAs in body fluids were widely reported as potential diagnostic, prognostic biomarker and therapeutic target in diverse tumors (74–76), including breast cancer (25), ovarian cancer (77), colorectal cancer (78), prostate cancer (79)., In particular, circulating miR-210 expression levels in tumor tissue and serum of ccRCC patients were detected by PCR and are consistently elevated compared to healthy controls (80). Recent study has demonstrated that miR-210 can be induced by hypoxia-inducible factor 1*α* (HIF-1*α*) to express in the tumor microenvironment, which correlates with unfavorable prognosis and greater resistance to chemo-radiotherapy in pancreatic cancer (81). Ahmed et al (82). found that serum miR-210 had 73.7% sensitivity and 64.28% specificity in discriminating hepatocellular carcinoma from other metastatic liver cancers, respectively. In addition, plasma miR-210 levels in breast cancer patients were related with sensitivity to trastuzumab, tumor presence as well as lymph node metastasis (83).

In summary, miRNAs have potential to be biomarkers for the diagnosis, therapy, and prognosis of ccRCC.

## Data Availability Statement

All datasets analyzed for this study are included in the article/**Supplementary Material**.

## Ethics Statement

This study has been approved by the Henan University institutional committee.

## Author Contributions

LX and XG perceived the conception and instructed the study. XM, FN, LZ, ZY, and HL searched and collected data. GC, ML, GZ, and YH performed the statistical analysis. GC and ML wrote the manuscript. All authors contributed to the article and approved the submitted version.

## Funding

This study is supported by Projects for College Students in Henan University (No. 2020101901), Program for Science and Technology Development in Henan Province (No. 162102310391, No.172102210187), Program for Scientific and Technological Research of Henan Education Department (No. 14B520022), Kaifeng Science and Technology Major Project (18ZD008), supporting grants of Henan University (No. 2015YBZR048; No. B2015151), and Program for Innovative Talents of Science and Technology in Henan Province (No. 18HASTIT048, No. 192102310379), supporting grant of Bioinformatics Center of Henan University (No. 2018YLJC01 and 2019YLXKJC04).

## Conflict of Interest

The authors declare that the research was conducted in the absence of any commercial or financial relationships that could be construed as a potential conflict of interest.

## Supplementary Material

Supplementary Figure 1 Meta-analysis of univariate Cox results reporting impact of miR-21 and miR-126 on survival in patients with ccRCC using random effects model.

Supplementary Figure 2 Functional enrichment analysis of the potential target genes in miR-155 **(A)**. Chart of the GO and KEGG enriched pathways **(B)**; Network of GO and KEGG enriched terms colored by clusters.

Supplementary Figure 3 Functional enrichment analysis of the potential target genes in miR-126 **(A)**. Chart of the GO and KEGG enriched pathways **(B)**; Network of GO and KEGG enriched terms colored by clusters.

Supplementary Figure 4 Functional enrichment analysis of the potential target genes in miR-141 **(A)**. Chart of the GO and KEGG enriched pathways **(B)**; Network of GO and KEGG enriched terms colored by clusters.

Supplementary Figure 5 Functional enrichment analysis of the potential target genes in miR-221 **(A)**. Chart of the GO and KEGG enriched pathways **(B)**; Network of GO and KEGG enriched terms colored by clusters.

## Abbreviations

CI, confidence interval; HR, hazard ratio.
