# Supplementary figures and images for: Systematic Analysis of microRNA Biomarkers for Diagnosis, Prognosis, and Therapy in Patients With Clear Cell Renal Cell Carcinoma
Source: Front Oncol. 2020 Dec 4;10:543817. doi: 10.3389/fonc.2020.543817 (PMC7746831; doi:10.3389/fonc.2020.543817)

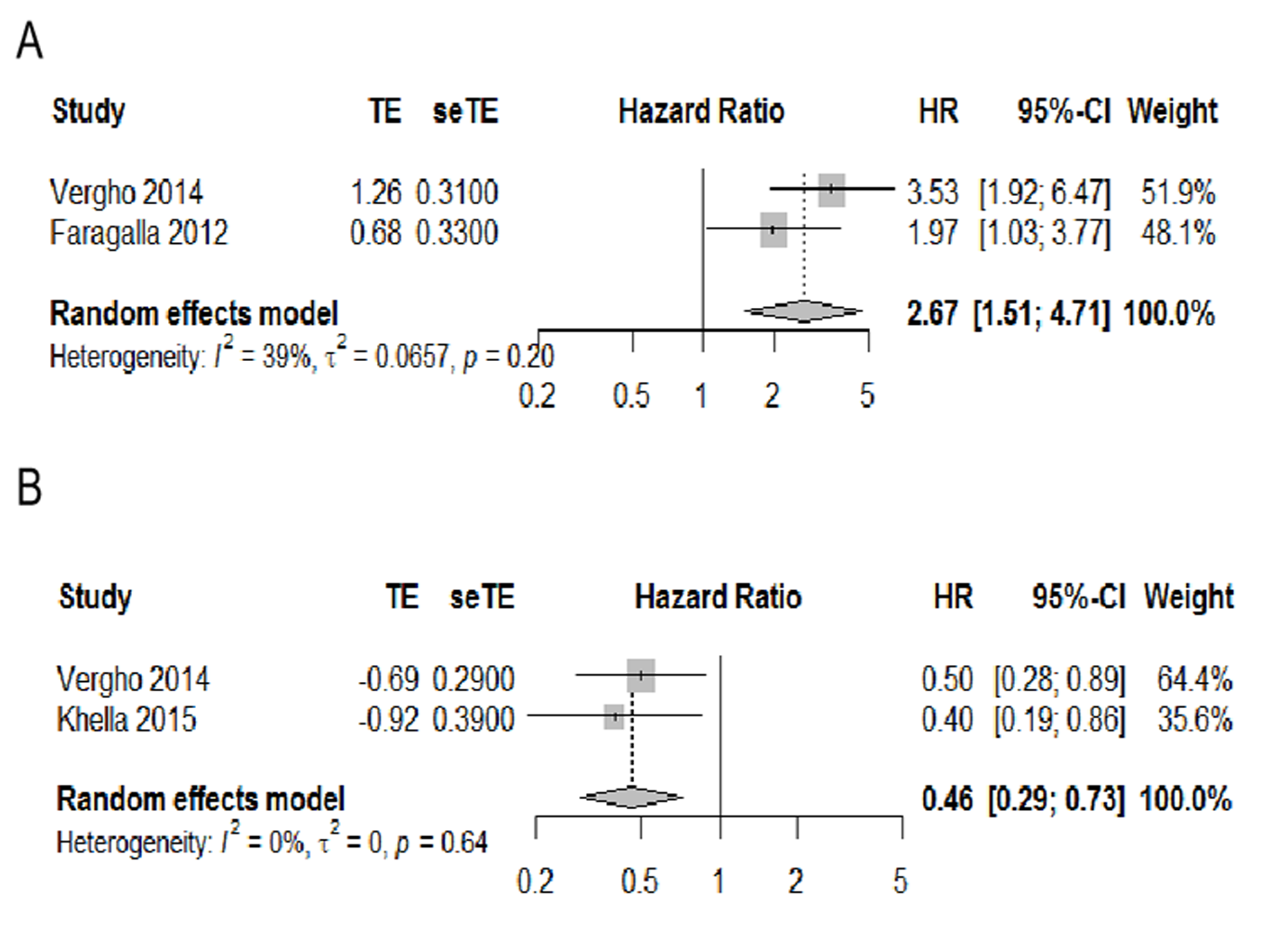

Supplement: Supplementary Figure 1 — Meta-analysis of univariate Cox results reporting impact of miR-21 and miR-126 on survival in patients with ccRCC using random effects model. [file Image_1.tif]

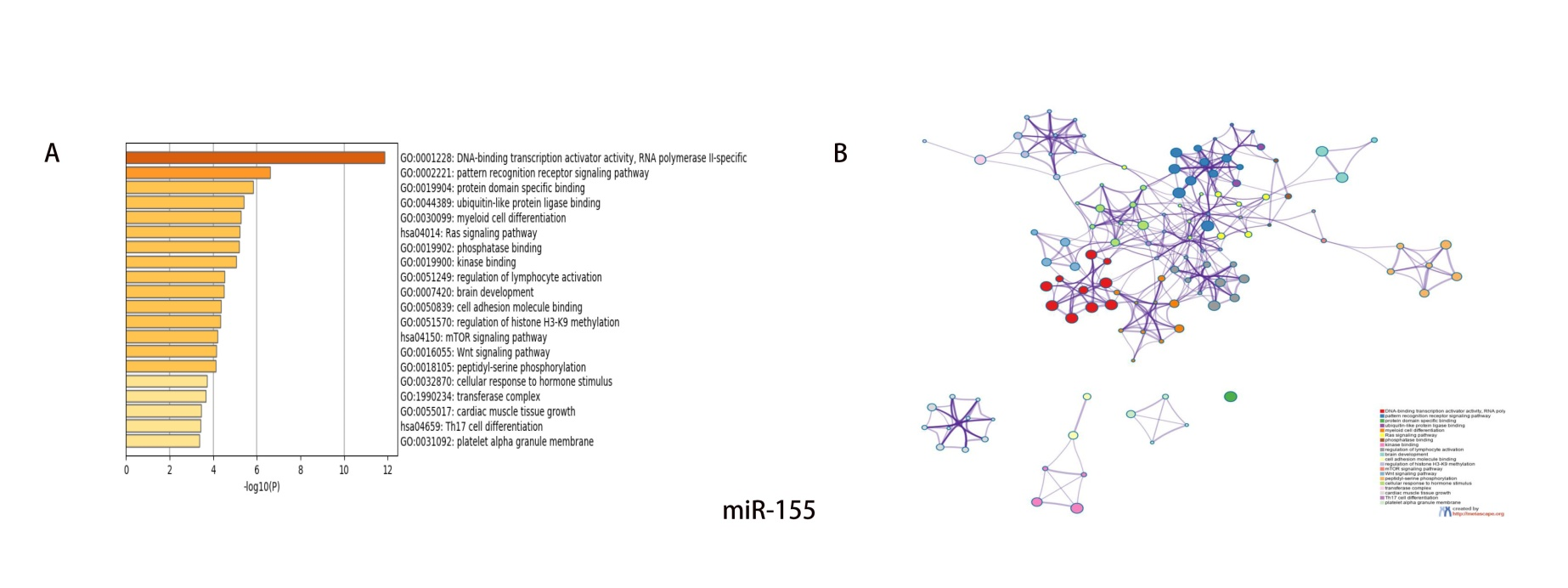

Supplement: Supplementary Figure 2 — Functional enrichment analysis of the potential target genes in miR-155 (A). Chart of the GO and KEGG enriched pathways (B); Network of GO and KEGG enriched terms colored by clusters. [file Image_2.tif]

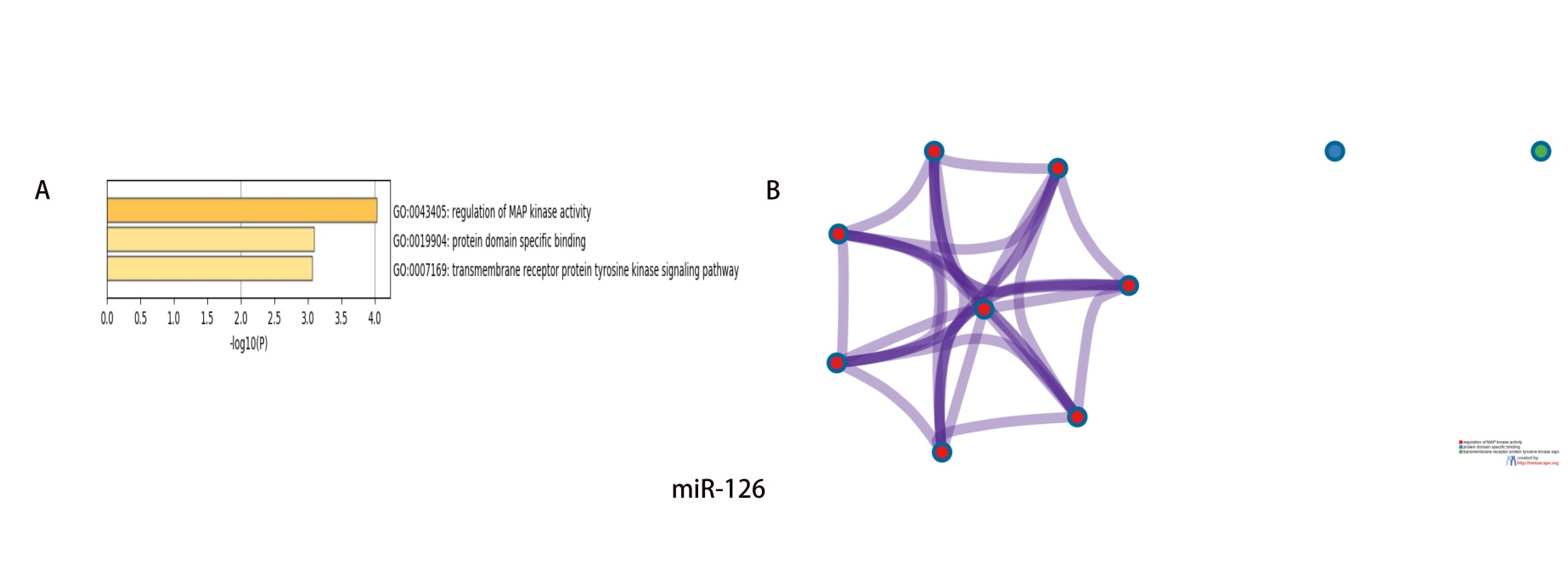

Supplement: Supplementary Figure 3 — Functional enrichment analysis of the potential target genes in miR-126 (A). Chart of the GO and KEGG enriched pathways (B); Network of GO and KEGG enriched terms colored by clusters. [file Image_3.tif]

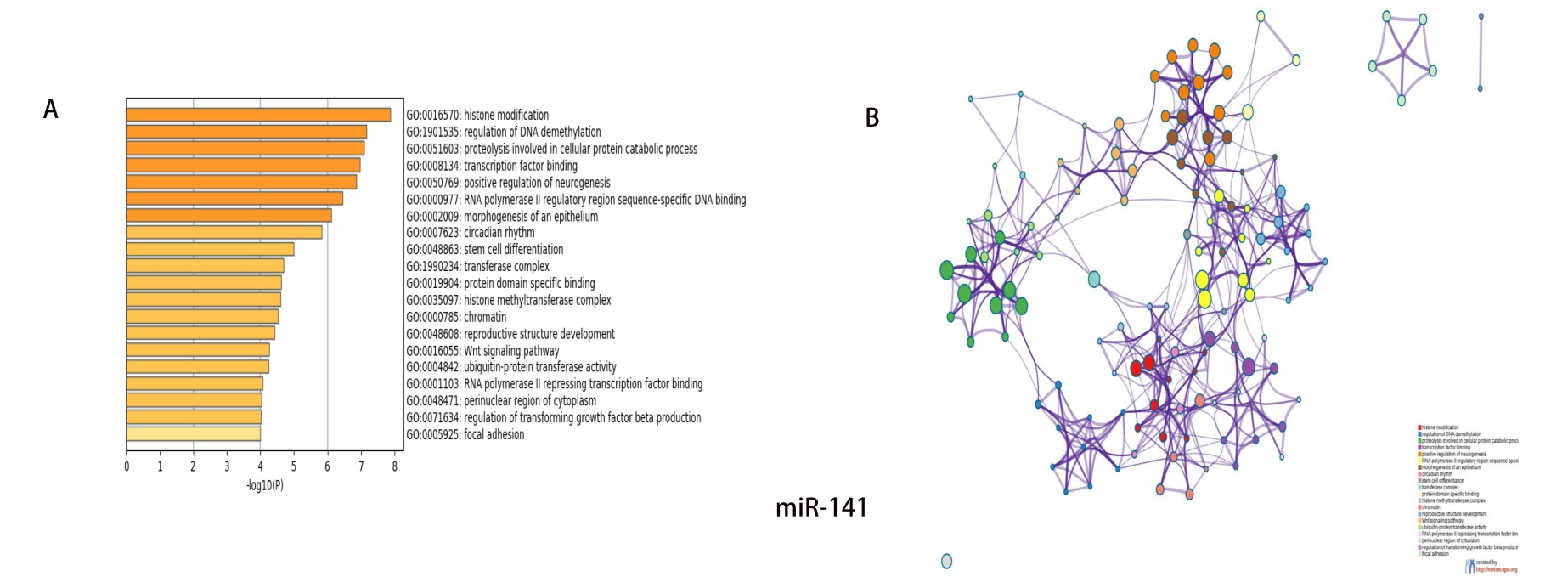

Supplement: Supplementary Figure 4 — Functional enrichment analysis of the potential target genes in miR-141 (A). Chart of the GO and KEGG enriched pathways (B); Network of GO and KEGG enriched terms colored by clusters. [file Image_4.tif]

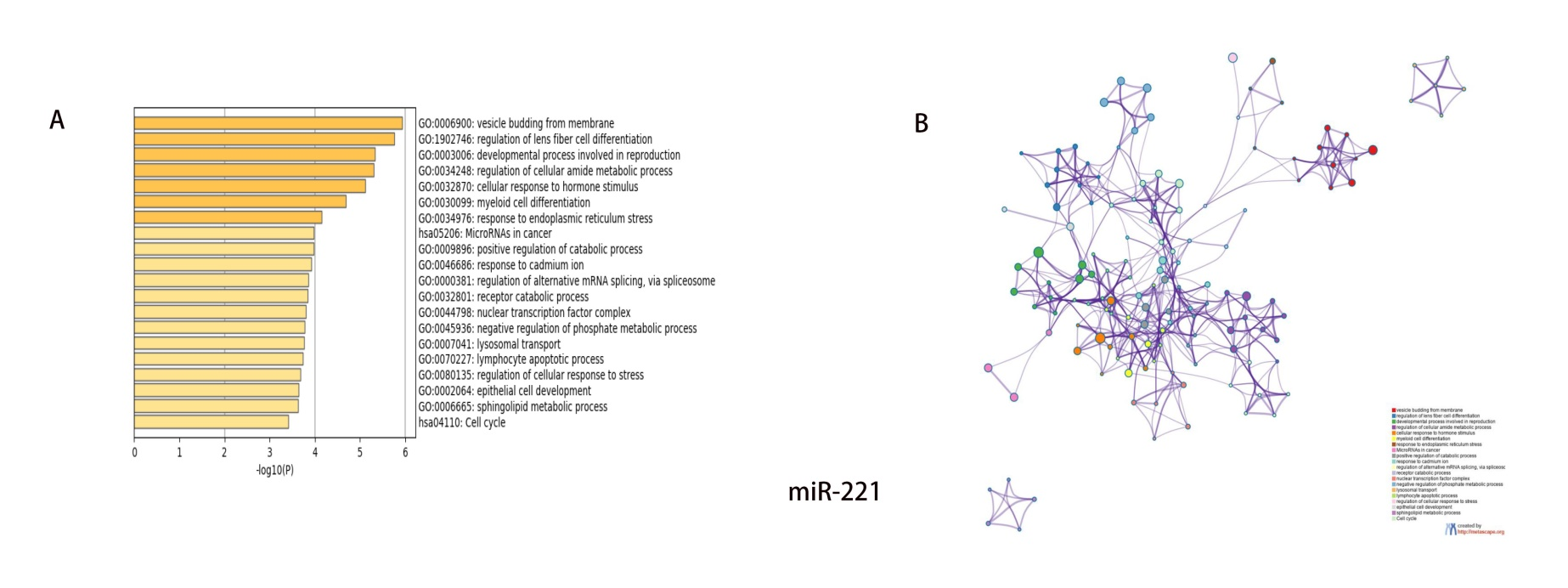

Supplement: Supplementary Figure 5 — Functional enrichment analysis of the potential target genes in miR-221 (A). Chart of the GO and KEGG enriched pathways (B); Network of GO and KEGG enriched terms colored by clusters. [file Image_5.tif]
