# Supplementary material for: Systematic Analysis of microRNA Biomarkers for Diagnosis, Prognosis, and Therapy in Patients With Clear Cell Renal Cell Carcinoma
Source: Front Oncol. 2020 Dec 4;10:543817. doi: 10.3389/fonc.2020.543817 (PMC7746831; doi:10.3389/fonc.2020.543817)
Supplement: Supplementary file 7 [file Table_2.docx]

**Table S2. The terms of populations, interventions, controls, outcomes, and study designs (PICOS) in this study.**

| Population | Clear cell renal cell carcinoma patients with gene expression information |
| --- | --- |
| Intervention | Expressing level of miRNAs in tissues and body fluids of ccRCC patients or high expression miRNAs ccRCC group |
| Control | Expressing level of miRNAs in tissues and body fluids of healthy people or low expression miRNAs ccRCC group |
| Outcome | Overall survival (OS), Disease free survival (DFS) and Recurrence free survival (RFS), Cancer specific survival (CSS), Metastasis free survival(MFS), Progression free survival(PFS), Disease specific survival(DFS) |
