# Supplementary material for: Systematic Analysis of microRNA Biomarkers for Diagnosis, Prognosis, and Therapy in Patients With Clear Cell Renal Cell Carcinoma
Source: Front Oncol. 2020 Dec 4;10:543817. doi: 10.3389/fonc.2020.543817 (PMC7746831; doi:10.3389/fonc.2020.543817)
Supplement: Supplementary file 8 [file Table_3.docx]

**Table S3. 118 ccRCC related miRNAs in human tissues as diagnostic biomarkers.**

| Name | Expression Level | n (ccRCC) | n (Control) | Sample Source | Detection Method | Target | PubMed ID |
| --- | --- | --- | --- | --- | --- | --- | --- |
| miR-21 | **Up** | 104 | 104 | Tissue | qRT-PCR | TIMP3 | 29131259 |
|  | **Up** | 24 | 24 | Tissue | qRT-PCR | ND | 27427222 |
|  | **Up** | 71 | 71 | Tissue | qRT-PCR | ND | 22580180 |
|  | **Up** | 28 | 28 | Fresh frozen | qRT-PCR | ND | 24647573 |
|  | **Up** | 25 | 25 | Tissue | qRT-PCR | CASC2 | 27222255 |
|  | **Up** | 32 | 32 | Fresh frozen | qRT-PCR | ND | 25381221 |
|  | **Up** | 30 | 10 | Tissue | qRT-PCR | ND | 26572589 |
|  | **Down** | 30 | 10 | Fresh frozen | qRT-PCR | ND | 24129247 |
| miR-155 | **Up** | 137 | 77 | Tissue | qRT-PCR | ND | 23050614 |
|  | **Up** | 30 | 10 | Fresh frozen | qRT-PCR | ND | 24129247 |
|  | **Up** | 78 | 78 | Fresh frozen | qRT-PCR | ND | 24647574 |
|  | **Up** | 32 | 32 | Fresh frozen | qRT-PCR | ND | 25381221 |
|  | **Up** | 36 | 36 | Fresh frozen | qRT-PCR | ND | 29228417 |
| miR-141 | **Down** | 30 | 10 | Fresh frozen | qRT-PCR | ND | 24129247 |
|  | **Down** | 78 | 78 | Fresh frozen | qRT-PCR | EphA2 | 24647573 |
| miR-221 | **Up** | 28 | 28 | Fresh frozen | qRT-PCR | TIMP2 | 26191221 |
|  | **Up** | 24 | 24 | Tissue | qRT-PCR | ND | 27427222 |
| miR-122 | **Up** | 32 | 32 | Fresh frozen | qRT-PCR | ND | 25381221 |
|  | **Up** | 148 | 60 | Fresh frozen | qRT-PCR | Dicer | 28921581 |
| miR-210 | **Up** | 78 | 78 | Fresh frozen | qRT-PCR | ND | 24647573 |
|  | **Up** | 32 | 32 | Fresh frozen | qRT-PCR | ND | 25381221 |
| miR-126 | **Up** | 78 | 78 | Fresh frozen | qRT-PCR | ND | 24647573 |
|  | **Down** | 128 | 128 | Tissue | qRT-PCR | ND | 27108693 |
| miR-362-3p | **Down**  **Down** | 36 | 36 | Fresh frozen  Fresh frozen | qRT-PCR  qRT-PCR | NLK  ND | 26647877  24647573 |
| miR-429 | **Down** | 96 | 96 | Tissue | qRT-PCR | ND | 31214494 |
|  | **Down** | 78 | 78 | Fresh frozen | qRT-PCR | ND | 24647573 |
| miR-509-5p | **Down** | 30 | 30 | Fresh frozen | qRT-PCR | ND | 23619562 |
|  | **Down** | 78 | 78 | Fresh frozen | qRT-PCR | ND | 24647573 |
| miR-7 | **Up** | 78 | 78 | Fresh frozen | qRT-PCR | ND | 24647573 |
|  | **Up** | 39 | 39 | Fresh frozen | qRT-PCR | ND | 23793934 |
| miR-142-3p | **Up** | 78 | 78 | Fresh frozen | qRT-PCR | ND | 24647573 |
|  | **Up** | 42 | 42 | Fresh frozen | qRT-PCR | ND | 26893725 |
| miR-30b | **Up** | 26 | 26 | Fresh frozen | qRT-PCR | ND | 28259953 |
|  | **Down** | 78 | 78 | Fresh frozen | qRT-PCR | ND | 24647573 |
| miR-377 | **Down** |  |  | Tissue | qRT-PCR | EST1 | 25776481 |
|  | **Down** | 78 | 78 | Fresh frozen | qRT-PCR | ND | 24647573 |
| miR-141-3p | **Down** | 27 | 27 | Fresh frozen | qRT-PCR | LOX | 27336447 |
|  | **Down** | 96 | 96 | Tissue | qRT-PCR | ND | 31214494 |
| miR-210-3P | **Up** | 96 | 96 | Tissue | qRT-PCR | ND | 31214494 |
| miR-142-5p | **Up** | 78 | 78 | Fresh frozen | qRT-PCR | ND | 24647573 |
| miR-122-5p | **Up** | 96 | 96 | Tissue | qRT-PCR | ND | 31214494 |
| miR-155-5p | **Up** | 96 | 96 | Tissue | qRT-PCR | ND | 31214494 |
| miR-21-5p | **Up** | 96 | 96 | Tissue | qRT-PCR | ND | 31214494 |
| miR-135a | **Down** | 78 | 78 | Fresh frozen | qRT-PCR | ND | 24647573 |
| miR-135a-5p | **Down** | 96 | 96 | Tissue | qRT-PCR | ND | 31214494 |
| miR-182 | **Down** | 57 | 57 | Fresh frozen | qRT-PCR | IGF1R | 27468875 |
| miR-30a-5p | **Down** | 96 | 96 | Tissue | qRT-PCR | ND | 31214494 |
| miR-30a | **Down** | 78 | 78 | Fresh frozen | qRT-PCR | ND | 24647573 |
| miR-30c-2 | **Down** | 78 | 78 | Fresh frozen | qRT-PCR | ND | 24647573 |
| miR-30c | **Down** | 78 | 78 | Fresh frozen | qRT-PCR | ND | 24647573 |
| miR-30d | **Down** | 78 | 78 | Fresh frozen | qRT-PCR | ND | 24647573 |
| miR-181d | **Up** | 78 | 78 | Fresh frozen | qRT-PCR | ND | 24647573 |
| miR-181b | **Up** | 78 | 78 | Fresh frozen | qRT-PCR | ND | 24647573 |
| miR-181a-5p | **Up** | 18 | 18 | Tissue | qRT-PCR | ND | 29693121 |
| miR-181c | **Up** | 78 | 78 | Fresh frozen | qRT-PCR | ND | 24647573 |
| miR-34a | **Up** | 78 | 78 | Fresh frozen | qRT-PCR | ND | 24647573 |
| miR-34b | **Up** | 78 | 78 | Fresh frozen | qRT-PCR | ND | 24647573 |
| miR-15a-5p | **Up** | 25 | 25 | Tissue | qRT-PCR | ND | 28098906 |
| miR-15a | **Up** | 78 | 78 | Fresh frozen | qRT-PCR | ND | 24647573 |
| miR-195-3p | **Up** | 26 | 26 | Tissue | qRT-PCR | ND | 28260025 |
| miR-195 | **Up** | 78 | 78 | Fresh frozen | qRT-PCR | ND | 24647573 |
| miR-200c-3p | **Down** | 96 | 96 | Tissue | qRT-PCR | ND | 31214494 |
| miR-200c | **Down** | 78 | 78 | Fresh frozen | qRT-PCR | ND | 24647573 |
| miR-510 | **Down** | 78 | 78 | Fresh frozen | qRT-PCR | ND | 24647573 |
| miR-510-5p | **Down** | 39 | 39 | Fresh frozen | qRT-PCR | ND | 25936999 |
| miR-146a-5p | **Up** | 16 | 22 | Fresh frozen | qRT-PCR | ND | 26859141 |
| miR-146a | **Up** | 78 | 78 | Fresh frozen | qRT-PCR | ND | 24647573 |
| miR-514 | **Down** | 78 | 78 | Fresh frozen | qRT-PCR | ND | 24647573 |
| miR-514a-3p | **Down** | 25 | 25 | Fresh frozen | qRT-PCR | ND | 29113192 |
| miR-452 | **Up** | 78 | 78 | Fresh frozen | qRT-PCR | ND | 24647573 |
| miR-1271 | **Up** | 78 | 78 | Fresh frozen | qRT-PCR | ND | 24647573 |
| miR-144 | **Up** | 78 | 78 | Fresh frozen | qRT-PCR | ND | 24647573 |
| miR-342-5p | **Up** | 78 | 78 | Fresh frozen | qRT-PCR | ND | 24647573 |
| miR-16-2 | **Up** | 78 | 78 | Fresh frozen | qRT-PCR | ND | 24647573 |
| miR-143 | **Up** | 78 | 78 | Fresh frozen | qRT-PCR | ND | 24647573 |
| miR-28-3p | **Up** | 78 | 78 | Fresh frozen | qRT-PCR | ND | 24647573 |
| miR-193a-5p | **Up** | 78 | 78 | Fresh frozen | qRT-PCR | ND | 24647573 |
| miR-193a-3p | **Up** | 78 | 78 | Fresh frozen | qRT-PCR | ND | 24647573 |
| miR-342-3p | **Up** | 78 | 78 | Fresh frozen | qRT-PCR | ND | 24647573 |
| miR-224 | **Up** | 78 | 78 | Fresh frozen | qRT-PCR | ND | 24647573 |
| miR-361-3p | **Up** | 78 | 78 | Fresh frozen | qRT-PCR | ND | 24647573 |
| miR-130b | **Up** | 78 | 78 | Fresh frozen | qRT-PCR | ND | 24647573 |
| miR-138 | **Down** | 78 | 78 | Fresh frozen | qRT-PCR | ND | 24647573 |
| miR-509-3p | **Down** | 78 | 78 | Fresh frozen | qRT-PCR | ND | 24647573 |
| miR-9 | **Down** | 78 | 78 | Fresh frozen | qRT-PCR | ND | 24647573 |
| miR-508-3p | **Down** | 78 | 78 | Fresh frozen | qRT-PCR | ND | 24647573 |
| miR-124 | **Down** | 78 | 78 | Fresh frozen | qRT-PCR | ND | 24647573 |
| miR-218 | **Down** | 78 | 78 | Fresh frozen | qRT-PCR | ND | 24647573 |
| miR-200b | **Down** | 78 | 78 | Fresh frozen | qRT-PCR | ND | 24647573 |
| miR-363 | **Down** | 78 | 78 | Fresh frozen | qRT-PCR | ND | 24647573 |
| miR-337-5p | **Down** | 78 | 78 | Fresh frozen | qRT-PCR | ND | 24647573 |
| miR-154 | **Down** | 78 | 78 | Fresh frozen | qRT-PCR | ND | 24647573 |
| miR-376a | **Down** | 78 | 78 | Fresh frozen | qRT-PCR | ND | 24647573 |
| miR-376c | **Down** | 78 | 78 | Fresh frozen | qRT-PCR | ND | 24647573 |
| miR-136 | **Down** | 78 | 78 | Fresh frozen | qRT-PCR | ND | 24647573 |
| miR-183 | **Down** | 78 | 78 | Fresh frozen | qRT-PCR | ND | 24647573 |
| miR-532-5p | **Down** | 78 | 78 | Fresh frozen | qRT-PCR | ND | 24647573 |
| miR-335 | **Down** | 78 | 78 | Fresh frozen | qRT-PCR | ND | 24647573 |
| miR-660 | **Down** | 78 | 78 | Fresh frozen | qRT-PCR | ND | 24647573 |
| miR-532-3p | **Down** | 78 | 78 | Fresh frozen | qRT-PCR | ND | 24647573 |
| miR-551b | **Down** | 78 | 78 | Fresh frozen | qRT-PCR | ND | 24647573 |
| miR-188-3p | **Down** | 78 | 78 | Fresh frozen | qRT-PCR | ND | 24647573 |
| miR-194 | **Down** | 78 | 78 | Fresh frozen | qRT-PCR | ND | 24647573 |
| miR-10a | **Down** | 78 | 78 | Fresh frozen | qRT-PCR | ND | 24647573 |
| miR-1280 | **Down** | 78 | 78 | Fresh frozen | qRT-PCR | ND | 24647573 |
| miR-362-5p | **Down** | 78 | 78 | Fresh frozen | qRT-PCR | ND | 24647573 |
| miR-502-5p | **Down** | 78 | 78 | Fresh frozen | qRT-PCR | ND | 24647573 |
| miR-10b | **Down** | 78 | 78 | Fresh frozen | qRT-PCR | ND | 24647573 |
| miR-378 | **Down** | 78 | 78 | Fresh frozen | qRT-PCR | ND | 24647573 |
| miR-629 | **Up** | 32 | 32 | Fresh frozen | qRT-PCR | TRIM33 | 25381221 |
| miR-185-5p | **Up** | 96 | 96 | Tissue | qRT-PCR | ND | 31214494 |
| miR-106b | **Up** | 26 | 26 | Fresh frozen | qRT-PCR | ND | 26648244 |
| miR-106a-5p | **Up** | 96 | 96 | Tissue | qRT-PCR | ND | 31214494 |
| miR-34b-3p | **Up** | 96 | 96 | Tissue | qRT-PCR | ND | 31214494 |
| miR-218-5p | **Down** | 96 | 96 | Tissue | qRT-PCR | ND | 31214494 |
| miR-200a-3p | **Down** | 96 | 96 | Tissue | qRT-PCR | ND | 31214494 |
| miR-200b-3p | **Down** | 96 | 96 | Tissue | qRT-PCR | ND | 31214494 |
| miR-584 | **Down** | 14 | 14 | Tissue | qRT-PCR | ROCK-1 | 21119662 |
| miR-200-b | **Down** | 30 | 10 | Fresh frozen | qRT-PCR | ND | 24129247 |
| miR-660-5p | **Down** | 25 | 25 | Fresh frozen | qRT-PCR | ND | 29138826 |
| miR-196a | **Down** | 48 | 48 | Fresh frozen | qRT-PCR | ND | 27175581 |
| miR-20b-5p | **Down** | 39 | 39 | Fresh frozen | qRT-PCR | ND | 26708577 |
| miR-149-5p | **Down** | 28 | 28 | Fresh frozen | qRT-PCR | ND | 27121091 |
| miR-539 | **Down** | 23 | 19 | Fresh frozen | qRT-PCR | ND | 29436648 |
| miR‐451a | **Down** | 15 | 15 | Tissue | qRT-PCR | ND | 29417701 |
| miR-129-3P | **Down** | 69 | 69 | Fresh frozen | qRT-PCR | ND | 24802708 |
| miR-125a-5p | **Down** | 39 | 39 | Tissue | qRT-PCR | ND | 25370896 |
| miR-217 | **Down** | 54 | 54 | Fresh frozen | qRT-PCR | ND | 23790169 |
| miR-99a | **Down** | 40 | 40 | Fresh frozen | qRT-PCR | mTOR | 23173671 |
| miR-187 | **Down** | 54 | 54 | Fresh frozen | qRT-PCR | B7-H3 | 23916610 |
| miR-186 | **Down** | 20 | 20 | Tissue | qRT-PCR | SENP1 | 28550686 |
| miR-506 | **Down** | 106 | 106 | Fresh frozen | qRT-PCR | FLOT1 | 25793370 |
| miR-100-5P | **Up** | 20 | 20 | Fresh frozen | qRT-PCR | ND | 28765937 |
| let-7 miRs | **Up** | 69 | 36 | Tissue | qRT-PCR | ND | 28694731 |
| miR-572 | **Up** | 39 | 39 | Fresh frozen and Formalin-ﬁxed parafﬁn-embedded | qRT-PCR | ND | 30132566 |
| miR-24-2 | **Up** | 28 | 28 | Fresh frozen | qRT-PCR | ND | 28990105 |
| miR-148a | **Down** | 52 | 52 | Fresh frozen | qRT-PCR | AKT2 | 27878305 |
| miR-145-5p | **Up** | 27 | 27 | Fresh frozen | qRT-PCR | LOX | 27336447 |
| miR-204 | **Down** | 65 | 65 | Fresh frozen | qRT-PCR | RAB22A | 26883716 |
| miR-128a-3p | **Up** | 16 | 22 | Fresh frozen | qRT-PCR /array | ND | 26859141 |
| miR-17-5p | **Up** | 16 | 22 | Fresh frozen | qRT-PCR /array | ND | 26859141 |

**Down: signiﬁcantly downregulated miRNAs in human ccRCC tissues; Up: signiﬁcantly upregulated miRNAs in human ccRCC tissues; qRT-PCR: quantitative real-time PCR; ND: not determined;**

**Abbreviations: TIMP3: TIMP metallopeptidase inhibitor 3; CASC2: cancer susceptibility 2; EphA2: erythropoietin-producing hepatocellularA2; TIMP2: TIMP metallopeptidase inhibitor 2; Dicer: dicer 1/ ribonuclease III; EST1: the transcription factor E26 transformation specific-1; IGF1R: insulin-like growth factor 1 receptor; LOX: lysyl oxidase; TRIM33: tripartite motif-containing 33; ROCK-1: rho associated coiled‑coil containing protein kinase 1; mTOR: mammalian target of rapamycin; B7-H3: B7 homolog 3; SENP1: sentrin-specific protease 1; FLOT1: flotillin 1; AKT2: AKT serine/threonine kinase 2; RAB22A:** **member RAS oncogene family; NLK: nemo like kinase.**
