# Supplementary material for: Systematic Analysis of microRNA Biomarkers for Diagnosis, Prognosis, and Therapy in Patients With Clear Cell Renal Cell Carcinoma
Source: Front Oncol. 2020 Dec 4;10:543817. doi: 10.3389/fonc.2020.543817 (PMC7746831; doi:10.3389/fonc.2020.543817)
Supplement: Supplementary file 9 [file Table_4.docx]

**Table S4. 28 ccRCC related miRNAs in human tissues as prognostic biomarkers**

| **Name** | **Expression Level** | **n (ccRCC)** | **Sample Source** | **Detection Method** | **Prognosis** | **Survival** | **PubMed ID** |
| --- | --- | --- | --- | --- | --- | --- | --- |
| miR-21 | Up | 37 | Formalin-ﬁxed parafﬁn-embedded | qRT-PCR | Unfavorable | CSS | 25279769 |
|  | Up | 71 | Tissue | qRT-PCR | Unfavorable | OS and DFS | 22580180 |
| miR-126 | Down | 264 | Tissue and Formalin-ﬁxed parafﬁn-embedded | qRT-PCR | Favorable | OS and DFS | 25572155 |
|  | Down | 37 | Formalin-ﬁxed parafﬁn-embedded | qRT-PCR | Favorable | CSS | 25279769 |
|  | Down | 116 | Formalin-ﬁxed parafﬁn-embedded | qRT-PCR | Favorable | DSS and RFS | 30988818 |
| miR-122 | Up | 46 | Tissue | qRT-PCR | Unfavorable | MFS | 30483771 |
|  | Up | 80 | Fresh frozen | qRT-PCR | Unfavorable | PFS | 28534944 |
| miR‐10a‐5p | Down | 202 | Formalin-ﬁxed parafﬁn-embedded | qRT-PCR | Favorable | CSS | 28432832 |
| miR-155 | Up | 137 | Tissue | qRT-PCR | Favorable | CSS and CFS | 23050614 |
| miR-221 | Up | 37 | Formalin-ﬁxed parafﬁn-embedded | qRT-PCR | Favorable | CSS | 25279769 |
| miR-203 | Down | 90 | Tissue | qRT-PCR | Unfavorable | OS | 25890121 |
| miR-200a | Down | 201 | Tissue | qRT-PCR | Unfavorable | OS | 27549611 |
| miR-200b | Down | 201 | Tissue | qRT-PCR | Unfavorable | OS | 27549611 |
| miR-200c | Down | 37 | Formalin-ﬁxed parafﬁn-embedded | qRT-PCR | Favorable | CSS | 25279769 |
| miR-210 | Up | 264 | Formalin-ﬁxed parafﬁn-embedded | qRT-PCR | Unfavorable | OS and CFS | 25555365 |
| miR-630 | Up | 92 | Tissue | qRT-PCR | Unfavorable | OS | 25031755 |
| miR-30a-5p | Down | 40 | Tissue | qRT-PCR | Favorable | OS | 28569782 |
| miR-497 | Down | 86 | Tissue | qRT-PCR | Favorable | OS | 25755771 |
| miR-27a-3p | Up | 159 | Tissue | qRT-PCR | Unfavorable | PFS and CSS | 26046464 |
| miR-217 | Down | 54 | Tissue | qRT-PCR | Favorable | OS | 23790169 |
| miR-3133 | Down | 135 | Tissue | qRT-PCR | Favorable | OS | 31192947 |
| miR-154-5p | Up | 17 | Formalin-ﬁxed parafﬁn-embedded | qRT-PCR | Unfavorable | OS | 30138594 |
| miR-663a | Up | 234 | Formalin-ﬁxed parafﬁn-embedded | qRT-PCR | Unfavorable | OS | 30021352 |
| miR-194 | Down | 234 | Formalin-ﬁxed parafﬁn-embedded | qRT-PCR | Favorable | OS and DFS | 26860079 |
| miR-29b | Up | 45 | Tissue | qRT-PCR | Unfavorable | OS | 26823729 |
| miR-129-3p | Down | 69 | Tissue | qRT-PCR | Favorable | OS and DFS | 24802708 |
| miR-144-3p | Down | 120 | Tissue | qRT-PCR | Favorable | OS | 27717821 |
| miR‐10b‐5p | Down | 202 | Formalin-ﬁxed parafﬁn-embedded | qRT-PCR | Unfavorable | CSS | 28432832 |
| miR-429 | Down | 201 | Tissue | qRT-PCR | Favorable | OS | 27619681 |
| miR-30a-3p | Down | 201 | Tissue | qRT-PCR | Favorable | OS | 27549611 |
| miR-30e | Down | 201 | Tissue | qRT-PCR | Favorable | OS | 27549611 |
| miR-30c | Down | 201 | Tissue | qRT-PCR | Favorable | OS | 27549611 |

**CSS: cancer specific survival; OS: overall survival; DFS: disease free survival; CFS: cancer-free survival; PFS: progression–free survival; DSS: disease-free survival; RFS: recurrence free survival; MFS: metastasis-free survival .**
