# Supplementary material for: Systematic Analysis of microRNA Biomarkers for Diagnosis, Prognosis, and Therapy in Patients With Clear Cell Renal Cell Carcinoma
Source: Front Oncol. 2020 Dec 4;10:543817. doi: 10.3389/fonc.2020.543817 (PMC7746831; doi:10.3389/fonc.2020.543817)
Supplement: Supplementary file 10 [file Table_5.docx]

**Table S5. Abnormally expressed miRNAs act as molecular targeted therapy**

| **Name** | **Expression Level** | **n (ccRCC)** | **n (control)** | **miRNA functions** | **Target** | **PubMed ID** |
| --- | --- | --- | --- | --- | --- | --- |
| miR-21 | Up | 99 | 99 | chemoresistance, migration, invasion | ND | 28714373 |
|  | Up | 25 | 25 | proliferation, migration | CASC2 | 27222255 |
|  | Up | 10 | 10 | proliferation，apoptosis， migration， invasion， EMT | ND | 29131259 |
| miR-122 | Down | 32 | 32 | proliferation, migration, invasion | Spry2 | 28231730 |
|  | Up | 148 | 148 | metastasis | Dicer | 28921581 |
|  | Up | 89 | 89 | migration, invasion | ND | 28534944 |
| miR-10b | Down | 262 | 262 |  | ND | 28360191 |
|  | Down | 9 | 9 | proliferation, cell-cycle, migration, invasion | ND | 26617769 |
| miR-106b-5p | Up | 8 | 8 | growth, metastasis | ND | 28423523 |
|  | Down | 40 | 40 | proliferation, apoptosis | ND | 25714014 |
| miR-497 | Down | 86 | 86 | proliferation, migration, invasion | SETD2 | 25755771 |
|  | Down | 40 | 40 | viability, apoptosis | VEGFR-2 | 28465356 |
| miR-200c | Down | 24 | 24 | docetaxel resistance | CYP1B1 | 25860934 |
|  | Down | 97 | 97 | proliferation, cell-cycle | CDK2 | 26248649 |
| miR-1 | Down | 40 | 40 | proliferation, invasion | TAGLN2 | 21745735 |
|  | Down | 44 | 44 | proliferation, motility | ND | 26036633 |
| miR-19a | Up | 70 | 70 | growth, invasion, migration | ND | 29474434 |
|  | Up | 18 | 18 | proliferation | PTEN/ SMAD4 | 27779660 |
| miR-203 | Down | 90 | 90 | proliferation, migration, invasion | FGF2 | 25890121 |
|  | Down | 24 | 24 | proliferation, migration, invasion, cell cycle arrest | HOTAIR | 29440295 |
| miR-126 | Up | 56 | 56 | proliferation | ND | 29202733 |
|  | Down | 128 | 128 | metastasis, proliferation, invasion, migration | ROCK1 | 27108693 |
| miR-30a-5p | Down | _ | _ | growth, migration and invasion, proliferation, metastasis, EMT, invasion | ND | 28569782 |
|  | Down | 593 | 389 | migration, invasion, proliferation | ND | 26002553 |
| miR-200c-3p | Down | 593 | 389 | migration, invasion, proliferation | ND | 26002553 |
| miR-23a-3p | Up | 20 | 20 | viability, mobility, apoptosis | PNRC2 | 30551118 |
| miR‐409‐3p | Down |  |  | hypoxia‐mediated glucose metabolism | ND | 30218446 |
| miR-210-3p | Down | 15 | 15 | metabolism, growth | TIGAR | 28138701 |
| miR-184 | Down | 50 | 50 | glucose consumption, lactate production, proliferation | PKM2 | 27431728 |
| miR-146a | Up | _ | _ | growth, malignant, epithelial–mesenchymal transition | CADM2 | 30184528 |
| miR-543 | Up | 20 | 20 | proliferation, invasion | KLF6 | 29101805 |
| miR-375 | Down | 27 | 27 | proliferation, migration, invasion | YWHAZ | 30082525 |
| miR-199a-3p | Down | 5 | 5 | invasion, migration, apoptosis , colony formation | ND | 29773428 |
| miR-320a | Down | 40 | 40 | viability, migration, invasion | FoxM1 | 30066895 |
| miR‑24‑2 | Up | 28 | 28 | proliferation, invasion, migration, apoptosis | ND | 28990105 |
| miR-599 | Down | 21 | 21 | proliferation, invasion | ND | 29568870 |
| miR-19b | Up | 70 | 70 | growth, invasion, migration | ND | 29474434 |
| miR-200b | Up | 56 | 56 | proliferation | ND | 29202733 |
| miR-93-3p | Up | 138 | 138 | proliferation, invasion, metastasis, apoptosis | PEDF | 29137305 |
| miR-144-3p | Up | 60 | 60 | proliferation, metastasis, chemoresistance | ND | 29073615 |
| miR-181a | Up | 42 | 42 | cell-cycle, apoptosis, proliferation | KLF6 | 29066014 |
| miR-367 | Up | 35 | 35 | proliferation, migration, invasion | MTA3 | 28968973 |
| miR‑373 | Up | 39 | 39 | proliferation | ND | 28901426 |
| miR-10a-5p | Down |  |  | proliferation, migration, invasion | ND | 28746769 |
| miR-1274a | Up | 40 | 40 | apoptosis, proliferation | BMPR1B | 29192325 |
| miR-23a-5p | Up | 24 | 24 | proliferation, apoptosis, viability, mobility | ND | 28656260 |
| miR-30e-3p | Down | 8 | 8 | invasion, migration | Snail1 | 28454361 |
| miR‑195‑3p | Up | 26 | 26 | apoptosis, mobility, proliferation | ND | 28260025 |
| miR‑30b | Up | 26 | 26 | proliferation, mobility, apoptosis | ND | 28259953 |
| miR-101 | Up | 25 | 25 | apoptosis, mobility | ND | 28098906 |
| miR‑15a‑5p | Up | 18 | 18 | proliferation | ND | 27779660 |
| miR-28-5p | Down | 33 | 33 | growth | RAP1B | 27729617 |
| miR-195 | Down | 30 | 30 | apoptosis, cell cycle arrest, migration, invasion, viability, lymph node metastasis | VEGFR2 | 27572273 |
| miR-141-3p | Down | 27 | 27 | migration, invasion | LOX | 27336447 |
| miR-145-5p | Down | 27 | 27 | migration, invasion | LOX | 27336447 |
| miR-196a | Down | 39 | 39 | migration proliferation, apoptosis | ND | 27175581 |
| miR-149-5p | Down | 28 | 28 | migration, proliferation, apoptosis | ND | 27121091 |
| miR-22 | Down | 68 | 68 | growth, migration, invasion | PTEN | 27082730 |
| miR-26a/b | Down | 15 | 15 | proliferation, migration, invasion | ND | 26983694 |
| miR-155 | Up |  |  | proliferation, motility, growth, invasion | E2F2 | 26967247 |
| miR-142-3p | Up | 42 | 42 | migration, proliferation, apoptosis | ND | 26893725 |
| miR-206 | Down | 41 | 41 | proliferation, proliferation | CDK4/CDK9/CCND1 | 26808577 |
| miR-451 | Down | 51 | 51 | apoptosis, growth | PSMB8 | 26779781 |
| miR-20b-5p | Down | 39 | 39 | migration, proliferation | ND | 26708577 |
| miR-362-3p | Down | 36 | 36 | proliferation, apoptosis, cell cycle, migration, invasion | NLK | 26647877 |
| miR‑429 | Down | 40 | 40 | Proliferation, migration, apoptosis | VEGF | 26647818 |
| miR‐490‐5p | Down |  |  | growth, migration, invasion | PIK3CA | 26559013 |
| miR-96 | Down | 63 | 63 | metastasis, invasion | Ezrin | 26419932 |
| miR‐372 | Down | 30 | 30 | Proliferation, invasion | IGF2BP1 | 26332146 |
| miR-221 | Up | 28 | 28 | proliferation, migration, invasion | TIMP2 | 26191221 |
| miR‐29a | Down | 24 | 24 | proliferation, migration, invasion | ND | 26096783 |
| miR‐29b | Down | 24 | 24 | proliferation, migration, invasion | ND | 26096783 |
| miR‐29c | Down | 24 | 24 | proliferation, migration, invasion | ND | 26096783 |
| miR-27a-3p | Up | 24(DC)/159(VC) | 24(DC)/159(VC) | proliferation, migration, invasion | ND | 26046464 |
| miR-124-3p | Down | 593 | 389 | cell cycle, proliferation | CAV1/FLOT1 | 26002553 |
| miR-506 | Down | 106 | 106 | migration, invasion | FLOT1 | 25793370 |
| miR-377 | Down | 18 | 18 | proliferation, migration, invasion | ETS1 | 25776481 |
| miR-107 | Down | 52 | 52 | cell cycle arrest，growth | EIF5 | 25758424 |
| miR-185 | Down | 40 | 40 | proliferation, apoptosis | VEGFA | 25700976 |
| miR-210 | Up | 40 | 40 | migration, proliferation, apoptosis | ND | 25555365 |
| miR‑451a | Up | 40 | 40 | proliferation, migration and apoptosis | ND | 25405789 |
| Let-7d | Down | 75 | 75 | growth, migration | ND | 25193015 |
| miR-646 | Down | 100 | 30 | metastasis, proliferation | NOB1 | 25010867 |
| miR‑145 | Down | 40 | 40 | proliferation, migration, invasion, apoptosis | ANGPT2/NEDD9 | 24384875 |
| miR‐143/145 cluster | Down | 18 | 18 | proliferation, invasion | HK2 | 24033605 |
| miR-187 | Down | 108 | 50 | migration, proliferation | ND | 23916610 |
| miR-217 | Down | 54 | 54 | proliferation, migration | ND | 23790169 |
| miR-192 | Down | 20 | 20 | invasion, proliferation, migration | ZEB2/MDM2/TYMS | 23715501 |
| miR-194 | Down | 20 | 20 | invasion, proliferation, migration | ZEB2/MDM2/TYMS | 23715501 |
| miR-215 | Down | 20 | 20 | invasion, proliferation, migration | ZEB2/MDM2/TYMS | 23715501 |
| miR-509-5p | Down | 30 | 30 | migration, apoptosis, proliferation | ND | 23619562 |
| miR‐135a | Down | 38 | 38 | proliferation, invasion | c‐MYC | 23176581 |
| miR-133a | Down | 40 | 40 | proliferation, invasion | TAGLN2 | 21745735 |

**DC: discovery cohort; VC: validation cohort;**

**Abbreviations: CASC2: cancer susceptibility 2; SPRY2: sprouty RTK signaling antagonist 2; SETD2: SET domain containing 2, histone lysine methyltransferase; VEGFR-2: Vascular Endothelial Growth Factor Receptor 2; CYP1B1: cytochrome P450 family 1 subfamily B member 1; CDK2：cyclin dependent kinase 2; CDK2：cyclin dependent kinase 2; PNRC2: proline-rich nuclear receptor coactivators; TIGAR：TP53 induced glycolysis regulatory phosphatase; PKM2: Pyruvate Kinase M2; CADM2: cell adhesion molecule 2; KLF6: Kruppel like factor 6; YWHAZ: tyrosine 3-monooxygenase/tryptophan 5-monooxygenase activation protein zeta; FOXM1: forkhead box M1; HOTAIR: HOX transcript antisense RNA; PEDF: Pigment Epithelium Derived Factor; KLF6: Kruppel like factor 6; YWHAZ: tyrosine 3-monooxygenase/tryptophan 5-monooxygenase activation protein zeta; FOXM1: forkhead box M1; HOTAIR: HOX transcript antisense RNA; KLF6: Kruppel like factor 6; MTA3: metastasis associated 1 family member 3; BMPR1B: bone morphogenetic protein receptor type 1; Snail1: snail family zinc finger 1a; SMAD4: SMAD family member 4; RAP1B: member of RAS oncogene family; Snail1: snail family transcriptional repressor 1; SMAD4: SMAD family member 4; RAP1B: member of RAS oncogene family; ROCK1: Rho associated coiled-coil containing protein kinase 1; E2F2: E2F transcription factor 2; FLOT1: flotillin 1; ETS1: ETS proto-oncogene 1, transcription factor; EIF5: eukaryotic translation initiation factor 5; VEGFA: vascular endothelial growth factor A; NOB1: NIN1 (RPN12) binding protein 1 homolog; ANGPT2: angiopoietin 2; NEDD9: neural precursor cell expressed, developmentally down-regulated 9; HK2: hexokinase 2; ZEB2: zinc finger E-box binding homeobox 2; MDM2: MDM2 proto-oncogene; TYMS: thymidylate synthetase; ZEB2: zinc finger E-box binding homeobox 2; MDM2: MDM2 proto-oncogene; TAGLN2: transgelin 2.**
