# Supplementary material for: Systematic Analysis of microRNA Biomarkers for Diagnosis, Prognosis, and Therapy in Patients With Clear Cell Renal Cell Carcinoma
Source: Front Oncol. 2020 Dec 4;10:543817. doi: 10.3389/fonc.2020.543817 (PMC7746831; doi:10.3389/fonc.2020.543817)
Supplement: Supplementary file 11 [file Table_6.docx]

**Table S6. The response of miRNAs after different drug treatments**

| Significant miRNA | Expression level | n ccRCC | Treatment result | Treatment method | PubMed ID |
| --- | --- | --- | --- | --- | --- |
| miR-99b-5p | Up | 90 | Long PFS | Sunitinib | 27738339 |
| miR-99b-5p | Up | 90 | Long PFS | TKI | 27738339 |
| miR-21 | Up | 28 | Sensitivity | MF | 26496641 |
| miR-144-3p | Up | 60 | Resistance | Sunitinib | 29073615 |

**TKI: antiangiogenic tyrosine kinase inhibitor; MF: metformin.**
